# Innovative approaches to bipolar disorder and its treatment

**DOI:** 10.1111/nyas.13048

**Published:** 2016-04-25

**Authors:** Paul J. Harrison, Andrea Cipriani, Catherine J. Harmer, Anna C. Nobre, Kate Saunders, Guy M. Goodwin, John R. Geddes

**Affiliations:** ^1^ David Geffen School of MedicineUniversity of CaliforniaLos Angeles; ^1^ Department of PsychiatryUniversity of OxfordOxfordUnited Kingdom; ^2^ Oxford Health NHS Foundation TrustOxfordUnited Kingdom; ^3^ Oxford Centre for Human Brain ActivityWarneford HospitalOxfordUnited Kingdom

**Keywords:** bipolar disorder, clinical, genetics, mood, therapy

## Abstract

All psychiatric disorders have suffered from a dearth of truly novel pharmacological interventions. In bipolar disorder, lithium remains a mainstay of treatment, six decades since its effects were serendipitously discovered. The lack of progress reflects several factors, including ignorance of the disorder's pathophysiology and the complexities of the clinical phenotype. After reviewing the current status, we discuss some ways forward. First, we highlight the need for a richer characterization of the clinical profile, facilitated by novel devices and new forms of data capture and analysis; such data are already promoting a reevaluation of the phenotype, with an emphasis on mood instability rather than on discrete clinical episodes. Second, experimental medicine can provide early indications of target engagement and therapeutic response, reducing the time, cost, and risk involved in evaluating potential mood stabilizers. Third, genomic data can inform target identification and validation, such as the increasing evidence for involvement of calcium channel genes in bipolar disorder. Finally, new methods and models relevant to bipolar disorder, including stem cells and genetically modified mice, are being used to study key pathways and drug effects. A combination of these approaches has real potential to break the impasse and deliver genuinely new treatments.

## Introduction

Bipolar disorder is classically described as clinically significant episodes of depression and elevated mood (mania or hypomania) with intervening periods of normal mood (euthymia).^1^ A distinction is made between type I and type II bipolar disorders that depends on the duration and severity of the episodes of mood elevation. In reality, the profile of bipolar disorder is complex and heterogeneous, both longitudinally and cross‐sectionally, and includes mixed mood states, persistent mood instability, and cognitive dysfunction.^2^, ^3^, ^4^, ^5^, ^6^, ^7^ During mood swings there may be features of psychosis (delusions and hallucinations) that are mood congruent. Although psychotic symptoms are seen only in a minority of patients, they explain the early terminology of *manic–depressive* psychosis. Psychotic symptoms also contribute to the uncertain position of bipolar disorder within psychiatric classifications that place it between schizophrenia and other mood disorders.^8^ The substantial morbidity of bipolar disorder arises primarily from the depressive episodes,^9^ and there is frequent comorbidity with anxiety disorders and substance misuse.^10^ Bipolar disorder affects 1–4% of the population, depending on the criteria used, with onset usually in adolescence or early adulthood.^11^ It is one of the leading causes of disability worldwide and is associated with significant direct and indirect costs.^12^ Suicide occurs in at least 5% of patients,^13^ and there is a marked increase in mortality rates from natural causes, especially cardiovascular disease.^14^ Consequently, life expectancy in bipolar disorder is reduced by 10 years or more.^15^, ^16^

## The current treatment of bipolar disorder

The prevalence of, morbidity from, and mortality and costs associated with bipolar disorder make its effective treatment and, ideally, prevention important goals within psychiatry. The following summary provides a brief overview of the current evidence and recommendations for treatment of bipolar disorder. Our discussion focuses on the results of network meta‐analyses, which are advanced statistical approaches to evidence synthesis that allow different interventions to be ranked for their relative effectiveness, even if they have not been compared in an individual trial^17^, ^18^ (for further review of bipolar disorder therapies see Ref. 19, and for recent clinical guidelines see Refs. 20, 21, 22).

## Evidence‐based treatment recommendations

The mainstay of therapy for all three phases of bipolar disorder (mania, depression, and prophylaxis) is pharmacological. The first‐line treatment for mania is an antipsychotic; inclusion of over 16,000 patients and 14 different treatments indicated that olanzapine and risperidone had the best profiles in terms of efficacy and tolerability.^23^ A subsequent update of the literature included several newer antimanic agents (notably cariprazine, a dopamine D_2_/D_3_ receptor partial agonist), but came to broadly similar conclusions.^24^

Bipolar depression is often long lasting and difficult to treat, requiring a different approach from that used in unipolar depression.^25^ The evidence regarding effective interventions is limited, and network meta‐analysis has reached inconsistent conclusions depending on how studies were included.^26^, ^27^ The broad consensus is that quetiapine, olanzapine, antidepressants, lamotrigine, and lurasidone have some efficacy but show varying tolerability. Relative efficacy is not well established by these analyses. Several recent clinical trials not included in these meta‐analyses provide new avenues for treatment of bipolar depression. Durgam *et al*.^28^ report efficacy of cariprazine (at 1.5 mg/day, but not at lower or higher doses) in a relatively large 8‐week trial. In a 12‐month double‐blind, placebo‐controlled, randomized trial, Geddes *et al*.^29^ showed that the combination of lamotrigine and quetiapine is more effective than quetiapine alone in patients with bipolar depression; unexpectedly, the benefit of lamotrigine was not seen in patients also randomized to folic acid. There is some evidence that the atypical antipsychotic lurasidone may have particular efficacy in bipolar depression with mixed features,^30^ and preliminary data support use of armodafinil as an adjunctive therapy.^31^, ^32^ Intravenous ketamine as an add‐on therapy to mood stabilizers shows potential to have a rapid but often transient antidepressant effect.^33^ Finally, a recent study highlights that electroconvulsive therapy remains a useful option for treatment‐resistant bipolar depression.^34^

For prevention of relapse in bipolar disorder, lithium remains the most effective and best studied monotherapy.^35^, ^36^ Comparison of lithium with other treatments is limited by the design of most relapse‐prevention studies, which are enriched for patients who have responded to the investigational drug for treatment of an episode of mania or depression.^37^ A network meta‐analysis of maintenance treatment was published in 2014, based on 33 trials involving 17 treatments or combinations and 6846 participants.^38^ This meta‐analysis included studies lasting at least 12 weeks with either a prophylaxis (where only euthymic participants were eligible) or a relapse‐prevention design (responders to the investigational drug during the acute phase were randomly assigned to either remain on the drug or be switched to placebo or comparator). The results support efficacy of a number of interventions, but only quetiapine and lithium prevented recurrence of both polarities of mood episode. Olanzapine, risperidone, and lithium in combination with valproate were significantly better than placebo in the prevention of manic episodes, and lamotrigine was better than placebo for depressive relapse. Valproate did not differ from placebo when depression and mania were considered separately. It is also noteworthy that, from a methodological viewpoint, the quality of the studies included in the meta‐analysis varied considerably, and these differences affected the final ranking of treatments. The efficacy of lithium was observed even when trial designs favored the active comparator. Hence, despite not being particularly well tolerated, lithium was supported as first‐line treatment; quetiapine, olanzapine, and lamotrigine were considered second line.^38^

Although the preceding discussion has focused on medication, because of their primary role in bipolar disorder and the number of new randomized clinical trials and meta‐analyses to highlight, psychological and psychosocial treatments, particularly lifestyle interventions, also play a role.^19^ In a recent systematic review,^39^ the authors concluded that the evidence is strongest for psychoeducation in the prevention of relapse in the early years after onset of bipolar disorder, with much more limited evidence for the use of cognitive behavioral and interpersonal therapies in the acute phases of the illness.

## Limitations of existing therapies

The preceding summary of evidence‐based guidelines and meta‐analyses emphasize that effective treatments for bipolar disorder are available. However, their effectiveness is modest, and all the drugs have significant side effects and potential harms.

Lithium's efficacy has to be balanced against its many side effects and potential toxicity. The risk of renal failure is a particular concern for both patients and clinicians. In fact, several recent studies show that this risk, though real, is considerably lower than often believed, especially if periods of acute lithium toxicity are avoided.^40^, ^41^, ^42^, ^43^, ^44^ There is an additional concern for women of childbearing age with bipolar disorder regarding pregnancy and breastfeeding. As well as a high risk of puerperal relapse,^45^ there are teratogenic and other risks to the fetus and baby associated with lithium and other mood stabilizers. Again, however, it is reassuring that the absolute pregnancy‐associated risks of lithium are not as great as previously thought,^40^, ^46^, ^47^ and some of the adverse pregnancy outcomes are related to bipolar disorder itself and not to its treatment.^48^ The risks of renal and other harms from lithium also have to be weighed against the strong evidence that it has an antisuicide effect^49^ and may also reduce risks of dementia,^50^ stroke,^51^ and overall mortality.^52^ Thus, in total, lithium appears to be a safer drug, when used judiciously, than usually considered.^53^, ^54^ Nevertheless, it is clearly associated with many side effects and risks, as are other mood stabilizers,^55^, ^56^, ^57^ and these limitations emphasize the need to develop new treatments for bipolar disorder which are more effective, tolerable, and safe.

Given the compelling need, why have there been no new drug treatments for bipolar disorder (other than repurposing of antipsychotics and anticonvulsants) since the introduction of lithium salts over 60 years ago? There are many reasons for this dearth of innovation. Most importantly, we do not have a good enough understanding of the pathophysiology of bipolar disorder, and therefore of rational drug targets for its treatment. The mechanisms of action of drugs currently used for bipolar disorder are unclear (in contrast to the better established pharmacological targets of antipsychotic, antidepressant, and anxiolytic drugs) and remain under active investigation. It is a paradox that lithium has one of the most specific therapeutic actions in psychiatry, yet has multiple different pharmacological and cellular effects.^58^ Prominent (and overlapping) hypotheses focus on lithium's inhibition of inositol monophosphatase and glycogen synthase kinase 3, and its effects on calcium signaling, mitochondrial function, and, more recently, neuroplasticity, neurogenesis, and G protein–activated potassium channels.^58^, ^59^, ^60^ The finding that two noncoding RNAs show genome‐wide association with lithium response may reveal additional targets and novel insight.^61^

A better understanding of the mechanism of action of lithium and other effective drugs should generate targets and aid new drug development. For example, the inhibition of inositol monophosphatase by lithium has led to evaluation of the putative lithium analogue ebselen, which shares this property. Ebselen has been found to have therapeutically relevant effects in animal models and in human subjects and is now proceeding into further development.^62^, ^63^ Although the mechanism of action of sodium valproate is unknown, it includes some of the effects noted for lithium in addition to having epigenetic effects via histone deacetylase inhibition.^64^, ^65^ For lamotrigine, the mechanism of action (at least in epilepsy) is thought to be via inactivation of presynaptic voltage‐gated sodium channels, and hence inhibition of glutamate release;^66^ however, it has many other actions which may be relevant to its role in bipolar disorder.^67^

Other reasons for the lack of effective innovation in bipolar disorder therapy include uncertainty about how best to define and determine therapeutic response, the lack of validated animal models (see below), and neglect in research funding compared to that for schizophrenia.^68^ Study of bipolar disorder poses additional problems because of the episodic nature of the condition, which requires long‐term studies to demonstrate prophylactic efficacy, as well as treatment trials for manic and depressive episodes. This scientific failure is of course not unique to bipolar disorder; it has been a problem across psychiatry and has contributed significantly to the recent withdrawal of many pharmaceutical companies from the field.^69^ Fortunately, the field may have reached a turning point, first, by taking advantage of novel ways to measure the clinical phenotype and the impact of a therapeutic intervention; and second, by building upon the advances in understanding of the etiology and pathogenesis of bipolar disorder that are emerging from genomics and from novel experimental approaches, such as genetic mouse models and human‐induced pluripotent stem cells (iPSCs). The remainder of this review summarizes recent progress in these areas.

## Refining the bipolar phenotype and how it is measured: a focus on mood instability

Psychiatric diagnoses are traditionally based on retrospective assessment of the history; follow‐up assessments are similarly based on the patient's account of the intervening weeks or months since the last appointment. Diagnosis has focused on the identification of “episodes,” and clinical outcome is often dichotomized, so that if a patient is not judged as reaching criteria for an episode, he/she is considered to be “well.”

These approaches are particularly problematic in bipolar disorder, wherein mood may fluctuate considerably—in either direction—during any time period evaluated in this way. The failure to measure symptom levels between episodes is a limitation because subsyndromal symptoms predict poor outcome and relapse.^70^, ^71^ One way to address this is to have more frequent and contemporaneous assessments of mood. In the recently completed CEQUEL trial of lamotrigine augmentation of quetiapine in bipolar depression,^29^ the primary outcome variable was the self‐report Quick Inventory of Depressive Symptoms,^72^ which was completed by participants remotely after a weekly text or e‐mail prompt. This approach had several advantages. First, it allowed subjects to be followed up relatively frequently and without requiring clinic visits beyond those required for their usual care––a valuable feature as trials become larger and longer. Second, weekly rating allows for a much more fine‐grained analysis of the response to treatment than just the prespecified time points at 12, 22, and 52 weeks, and reveals effects of lamotrigine beyond simply its antidepressant action (unpublished observations). In a separate study, analysis of daily mood ratings collected via a smartphone app showed a clear distinction in mood variability (as well as mood symptoms) between subjects with bipolar disorder and those with borderline personality disorder.^73^

The use of novel technologies can not only help us to capture mood and other mental state data more efficiently and accurately, but can move bipolar disorder research beyond our reliance on psychopathology to capture physiological, behavioral, and environmental data in order to identify the biological correlates and ultimately the underlying processes. Such data capture is increasingly feasible through the capabilities of smartphones, smartwatches, and wearable devices, and can include actigraphy, posture, GPS position, heart rate, temperature, and other factors. Many of these data can be acquired automatically, without requiring any action on the part of the subject, while others require their input. For example, in ongoing studies, we and others are using smartphones, wrist‐worn devices, and skin patches to capture data on physical activity, heart rate, and sleep, as well as delivering bespoke tests of cognitive function and emotional processing via apps on smartphones or tablets.^74^ These uses of remote technologies to augment treatment trials in bipolar disorder complement their rapid––though still largely untested and unregulated––implementation into routine clinical monitoring and self‐monitoring.^75^, ^76^, ^77^ In these respects, bipolar disorder is at the forefront of the big data revolution in health care. However, considerable further work is required to demonstrate the validity,^78^, ^79^, ^80^ feasibility,^81^, ^82^ and acceptability^83^ of these devices and approaches.

## The significance of mood instability in bipolar disorder

These issues can be well illustrated by reference to the investigation of mood instability, which, as we noted earlier, is a common feature of bipolar disorder, despite the textbook view that the disorder is one of discrete mood episodes interspersed with normal (and stable) mood. Although the presence of persistent mood instability is in fact well known to experienced clinicians and demonstrable using conventional methods,^84^, ^85^ remote monitoring and multidimensional data capture facilitate a more quantitative assessment and can be coupled to sophisticated mathematical techniques for data analysis.^86^, ^87^, ^88^ In addition to being a clinical feature of bipolar disorder, there is increasing evidence that mood instability is a symptom that is relatively common in the general population and a risk factor for a number of illness outcomes. Thus, it occurs in those at high risk for bipolar disorder^89^ and predicts its onset,^90^ it occurs during the prodrome of the disorder,^7^, ^91^ and it is independently associated with poor prognoses.^92^, ^93^, ^94^ Mood instability also contributes to borderline personality disorder and attention‐deficit disorder phenotypes.^5^

Given these considerations, research into mood instability will benefit from better definition^95^, ^96^ and improved understanding of its neural, molecular, and genetic bases.^97^ Indeed, there is an iterative process whereby the need to characterize, quantify, and understand mood instability and its correlates drives the development of devices and methods to achieve this, while the capabilities provided by the developments enhance the focus on the phenomenon and its measurement. For example, advances in neuroimaging methods and analysis tools allow investigation of mood instability and its relationship to variation in cognition, brain activity, and neural dynamics. By looking at patterns of correlation among signals across different brain areas, it is possible to reveal the functional networks,^98^ with activity in these networks varying dynamically as individuals perform psychological tasks or are at rest.^99^ By measuring brain activity at high temporal resolution using techniques such as magnetoencephalography,^100^ it becomes possible to measure the fluctuating dynamics across brain networks as they unfold.^101^, ^102^ Other methods identify the functional networks that are most active at any given time point^103^ and make it possible to derive measures of neural instability, and thereby to investigate what instabilities in neural processing may underpin cognitive and mood instability at various time scales. These approaches afford a new dimension to investigations of the neural bases of psychological disorders linked to mood instability, potentially revealing differences in the dynamics in brain networks linked to mood or cognition or differences in their regulation by executive control or reward‐related functions.

A combination of these and other new methods may also allow identification of predictive markers for the effects of mood‐stabilizing therapies and development of experimental medicine models for testing potential new bipolar disorder therapies. For example, lithium may affect mood instability or its cognitive and neural correlates independent of, and earlier than, its established efficacy against clinical episodes of mania or depression. To test this hypothesis, we are exploring the effects of first exposure to lithium on the variability of mood, neural response and networks, and cognitive function (focused on reward‐based decision making, learning, and attention), in a double‐blind, placebo‐controlled study.^74^ An effect of lithium on one or more metrics of variability will help identify biomarkers that can be used to test novel candidate mood stabilizers more rapidly than is the case using traditional randomized controlled trial designs. By reducing the time and thereby the costs and risks involved, an experimental medicine model of bipolar disorder would encourage reinvestment in the field. A precedent for such a model is provided by the conceptually equivalent discovery of cognitive and emotional biomarkers predictive of antidepressant efficacy in unipolar depression.^104^ The successful identification and validation of these markers is now used to inform and refine decision making about novel putative antidepressant medications.^105^

In summary, mood instability is of interest and potential importance in bipolar disorder in its own right. It also illustrates the novel conceptual and technical approaches that are being taken to characterize and understand the bipolar phenotype. In principle, the same rationale and multidisciplinary approaches can be applied to other features, such as reward sensitivity^106^ and sleep and circadian rhythm dysregulation (see below).

## Better understanding of etiology and pathophysiology

Although improving the measurement and clinical characterization of the bipolar disorder phenotype can facilitate more powerful and rapid identification of the effects of potential new treatments, transformative advances in therapy will require a substantially better understanding of the biological basis of the disorder. This, in turn, requires additional knowledge and novel tools. Fortunately, progress has been made in several areas, including genetics, animal models, and cellular models.

## Therapeutic potential of bipolar disorder genetics

Bipolar disorder has a high heritability (over 80%), with a complex non‐Mendelian genetic basis.^107^ The majority of genetic risk is associated with multiple polymorphisms, with a very small contribution from copy number variants and other rare variants.^107^, ^108^, ^109^, ^110^ The leading bipolar disorder loci and genes based on existing genome‐wide association studies (GWAS) are summarized in Table 1; many more will emerge with a forthcoming much larger assembly of data from GWAS from the Psychiatric Genomics Consortium.^108^ As with other diseases, genetic information has the potential to inform and improve bipolar disorder treatments, both by highlighting targets and pathways and by enabling personalized medicine.^107^ However, the magnitude and immediacy of such effects are limited because of the complexities of the genetic architecture and the many steps that lie between identification of a genetic locus and validation of a drug target.^111^, ^112^, ^113^, ^114^, ^115^, ^116^

**Table 1:** Genome‐wide significant bipolar disorder risk loci, implicated genes, and their therapeutic potential

Locus	Gene symbol(s)	Gene name(s)	Therapeutic potential^a^
10q21.2	*ANK3*	Ankyrin 3 (encodes ankyrin‐G)	++
12p13.3	*CACNA1C*	Voltage‐dependent calcium channel, L‐type, α1C (encodes Ca_v_1.2)	++++
11q14.1	*TENM4* b	Teneurin transmembrane protein 4	+
19p12	*NCAN*	Neurocan	++
5p15.31	*ADCY2*	Adenylate cyclase 2	+++
3p22.2	*TRANK1*	Tetratricopeptide repeat and anykrin repeat containing 1	+
10q24.33	*AS3MT*	Arsenite methyltransferase	++
6q25.2	*SYNE1*	Spectrin repeat containing, nuclear envelope 1	+
6q16.1	*MIR2113/POU3F2* c, d	MicroRNA 2113/POU class 3 homeobox 2	+
16p11.2	*MAPK3* e	Mitogen‐activated protein kinase 3	++
2q32.1	*ZNF804A* e	Zinc finger protein 804A	+
3p21.1	*ITIH3/ITIH4* c, e	Inter‐α‐trypsin inhibitor heavy chains 3 and 4	+
3p21	*PBRM1* f	Polybromo 1	+

a Rated from + (gene function unknown or unlikely to be druggable based on known biology) to ++++ (relevant biology and already targeted by licensed drugs for other indications).

b Also known as *ODZ4*.

c Locus of association lies between these genes.

d *POU3F2* is also known as *OTF7*.

e Genome‐wide significant in combined bipolar disorder and schizophrenia sample.

f Genome‐wide significant in combined bipolar disorder and major depression sample.

An abnormality of calcium signaling has long been considered a potential pathophysiological mechanism in bipolar disorder, based mostly on biochemical data in peripheral blood cells.^117^, ^118^, ^119^ It is therefore noteworthy that calcium channel genes are prominent in the genomic data.^107^, ^113^ The evidence is threefold. *CACNA1C*, which encodes the Ca_v_1.2 subunit of L‐type voltage‐gated channel, is one of the genes most robustly identified by GWAS; second, the functional category of calcium signaling is enriched among bipolar disorder–associated genes; and third, rare variants in calcium channel subunits are also implicated.^107^, ^118^, ^120^ The involvement of calcium channel genes in bipolar disorder is not only significant in terms of prior pathophysiological findings but because some data suggest that calcium channel antagonists (used to treat hypertension and angina) may have a role in bipolar disorder treatment.^121^ However, the findings are inconclusive, with randomized clinical trial data limited to small trials of verapamil for mania.^24^, ^122^ Nevertheless, the recent genetic data provide impetus to further investigate the role of L‐type calcium channel antagonists in bipolar disorder treatment; trials using these agents in bipolar disorder can now select or stratify participants based on *CACNA1C* risk genotype.^123^ Compared to verapamil, other drugs in this class have properties that may be advantageous in bipolar disorder, such as improved brain penetration, longer half‐life, and greater L‐type calcium channel subunit selectivity. Looking ahead, the ideal L‐type calcium channel antagonist for bipolar disorder would have specificity for isoforms that are preferentially expressed in the brain, compared to those expressed in the heart and blood vessels, in order to maximize efficacy and minimize cardiovascular side effects.^122^, ^124^

Several of the other genes listed in Table 1 also have potential as drug targets, although it may prove difficult to exploit these leads.^125^ For example, ankyrin G (encoded by *ANK3*) is involved in coupling voltage‐gated sodium channels to the axonal cytoskeleton.^126^ At first sight, this suggests a potential therapeutic role in regulation of neuronal excitability; but recent studies emphasize the complexity and diversity of ankyrin G distribution and function, and it is not clear which aspects are most relevant to bipolar disorder.^127^, ^128^ It is also not known what impact the *ANK3* risk variants have upon gene regulation or function, and therefore whether a drug targeting this gene product should enhance, inhibit, or stabilize ankyrin G activity.^129^

Genome‐wide association studies also confirm that bipolar disorder is not a discrete entity, genetically speaking. That is, much of the genetic risk for bipolar disorder is shared with schizophrenia, and a lesser but still significant amount with major depression, complementing the phenotypic overlaps and comorbidities known to every clinician.^8^, ^130^ There is also evidence, albeit less robust, for genetically distinct subgroups within bipolar disorder, for example in terms of the nature of psychotic or manic symptoms.^131^, ^132^, ^133^ Genetics is thereby contributing to the current interest in reconceptualizing psychiatric disorders, such as bipolar disorder, both transdiagnostically and in terms of their underlying biology, most prominently by the National Institute of Mental Health Research Domain Criteria initiative.^134^ This reformulation has therapeutic implications, encouraging a search for treatment targets and mechanisms that similarly cross conventional diagnostic boundaries. For bipolar disorder, these might include attentional or cognitive impairments^2^, ^7^, ^135^, ^136^ and, as noted earlier, mood instability independent of depressive or manic episodes. It might also include treatments to normalize sleep and circadian rhythms, with increasing evidence that such abnormalities are not just part of its symptomatology but may contribute to its onset and maintenance.^137^, ^138^, ^139^, ^140^, ^141^ Given these considerations, it is interesting that *CACNA1C* and other calcium channel genes also show genome‐wide association with sleep quality^142^, ^143^ and aspects of memory,^144^, ^145^ in addition to their role in risk for bipolar disorder and other psychiatric disorders.

## Better experimental methods to model bipolar disorder and its treatment

Along with genomics, cellular and animal models are crucial components of the target identification and drug discovery processes for many diseases. Both have been used in a number of studies in bipolar disorder, with interesting, though modest, findings.

### Cellular models

Existing data from bipolar disorder cellular models have recently been systematically reviewed.^146^ Most data come from studies using peripheral cells, and hence have inherent limitations (because they are non‐neuronal; and in the specifc case of lymphoblasts because they have undergone viral transformation). Moreover, many positive findings have not been replicated and their interpretation is unclear. Nevertheless, as mentioned earlier, these *in vitro* approaches have provided considerable evidence in bipolar disorder for abnormalities affecting calcium signaling, as well as alterations in mitochondrial function, apoptosis, and the circadian system. Abnormalities are generally greater in the presence of cellular stressors than at baseline, and are often normalized by lithium treatment. Reassuringly, some of the *in vitro* findings are complemented by similar findings in postmortem brain, and together provide some clues for novel therapeutic targets.^147^

Most ongoing *in vitro* medical research now uses iPSCs and cell reprogramming technologies to produce (directly or indirectly) neural precursors, neurons of various types, and even brain organoids. Bipolar disorder is no exception to these significant research advances, although data thus far remain limited and results modest.^148^, ^149^, ^150^, ^151^, ^152^ With regard to therapy, three recent papers are pertinent. Yoshimizu *et al*.^152^ studied neurons induced from subjects genotyped for the main bipolar disorder risk polymorphism in *CACNA1C* to examine the expression and function of calcium channels. Neurons derived from subjects homozygous for the risk variant expressed more *CACNA1C* mRNA and showed enhanced current density, compared to heterozygotes and nonrisk homozygous subjects. These results suggest that the risk variants of *CACNA1C* involve a gain of function (see also Ref. 153), and thus strengthen the case, discussed earlier, that L‐type calcium channel antagonists might be potential therapeutic agents in bipolar disorder. An alternative experimental design is to compare cells derived from drug‐responsive versus nonresponsive patients and to help identify the key molecular pathways and processes that may underlie therapeutic responsiveness *in vivo*. Using this approach, neurons induced from fibroblasts taken from lithium responders were found to have greater adhesiveness than those from nonresponders.^148^ Mertens *et al*.^151^ also showed a different molecular and functional profile of induced neurons from bipolar disorder patients according to their lithium responsiveness *in vivo*; notably, their data implicated mitochondrial and calcium signaling abnormalities, in line with the earlier data of this kind. The indications from iPSC studies that lithium responsiveness may reflect a pathophysiologically meaningful subtype of bipolar disorder complement the increasing evidence that it also delineates a clinically identifiable subtype of the disorder,^154^ for example, in terms of symptom profile and family history, and hence the potential value of biomarkers predictive of lithium response.^155^

Although these and other findings using reprogrammed cells are very preliminary, the rapid technical developments in the field promise significant advances and increases of scale in the near future, and the methods are likely to play a central role in target validation and drug discovery for bipolar disorder, as well as in the understanding of its etiology and pathophysiology.^156^

### Mouse models

The value of rodents for modeling psychiatric disorders and advancing treatment has been increasingly questioned.^157^ This applies both to genetic modifications and to phenotypes produced by pharmacological or behavioral interventions (e.g., amphetamine sensitization, isolation rearing). The problem is even greater for bipolar phenotype than, for example, for schizophrenia, since the animal model ideally needs to recapitulate spontaneous fluctuation between states––its definitive characteristic––as well as exhibit depressive‐like, manic‐like, and psychotic‐like phenotypes.^158^ However, progress is being made, partly due to a shift in views about the bipolar phenotype and what is being modeled,^159^ and partly due to technical advances in genetic and neural circuit manipulation of rodents (for recent examples, see Refs. 160, 161, 162, 163).

Perhaps the best known genetic mouse model relevant to bipolar disorder is the *Clock*Δ*19* mouse. This mouse strain has a mutation in the gene encoding CLOCK, a key regulator of the circadian system,^164^ and exhibits a characteristic diurnal behavioral profile of manic‐like (i.e., hyperactive) activity, with more reward‐related and less anxious or depressive features during the light phase, but normal behavior in the dark. The manic‐like behavior coincides with, and is at least partly caused by, increased firing of midbrain dopaminergic neurons, as shown using an optogenetic approach.^162^ Another circadian protein (the nuclear receptor REV‐ERBα) is involved in similar mood‐related behaviors and also regulates dopamine.^163^ Such findings argue for a renewed focus on dopamine, and on circadian rhythms, in the phenotype of bipolar disorder and potentially as targets for treatment.

These examples show how genetically modified mice are being used to investigate cellular and molecular mechanisms contributing to bipolar disorder–relevant phenotypes, even though the genes concerned, *Clock* and *Nr1d1* (encoding REV‐ERBa), do not currently show strong genetic association with bipolar disorder itself. A complementary approach is to study mice in which a manipulated gene locus does show genome‐wide association with bipolar disorder. For example, Leussis *et al*.^161^ investigated ANK3 (Table 1) by examining heterozygous *Ank3^+/–^* mice and by knocking down *Ank3* selectively in the dentate gyrus using RNA interference. Both manipulations led to reduced anxiety and increased reward motivation compared to wild‐type mice. The *Ank3^+/–^* mice also showed greater stress reactivity, developing more depression‐like behaviors and enhanced corticosterone levels after chronic stress. Furthermore, the phenotypes were normalized by chronic administration of lithium.

## Conclusions

Bipolar disorder exemplifies the challenges and the opportunities faced by psychiatry as it attempts, belatedly, to move forward from descriptive psychopathology and serendipitously discovered therapies of limited efficacy and tolerability to a more valid nosology and treatments that are based on rational understanding of pathophysiology, the latter requiring advances in molecular genetics and neuroscience. Althoug history cautions us to be prudent and not expect fundamental breakthroughs to be imminent, recent developments across a range of disciplines have permitted real optimism. Our discussion above has highlighted several developments: novel approaches to how the bipolar phenotype is conceptualized and measured and the prospects of linking this phenotype mechanistically to underlying genetic, molecular, and neural circuits. A range of new technologies (from remote biosensors to reprogrammed cells and optogenetics) and approaches (from big data to mathematical modeling and experimental medicine) are driving these developments. In addition to invigorating psychiatric research and bringing cutting‐edge neuroscientists and other disciplines to bear on these complex problems, these innovative approaches should encourage the pharmaceutical industry, other commercial partners (including device and software manufacturers), and funding bodies to invest in the field. The personal burden and substantial costs of bipolar disorder––to patients, families, and society––together with the unsatisfactory state of current interventions and outcomes, provide additional motivation to finally break the impasse regarding how the disorder is understood and treated. Input is also required from patients themselves: research needs their full involvement and engagement, both through participation and in advocacy. They know only too well the limitations of current treatments.

## Conflicts of interest

In the past 2 years, P.J.H. has served as an expert witness on patent litigation involving drugs used to treat bipolar disorder. A.C. has served as an expert witness for a patent litigation case involving quetiapine. C.J.H. has received consultancy fees from Lundbeck and P1vital, is a shareholder and company director of Oxford Psychologists Ltd., and has received research funding from UCB, J&J, Lundbeck, and Sunovion. G.M.G. holds shares in P1vital and has served as consultant, advisor, or speaker for AstraZeneca, Abbvie, Cephalon/Teva, Convergence, Eli Lilly, GSK, Lundbeck, Medscape, Merck, Otsuka, P1vital, Servier, Sunovion, and Takeda. The other authors report no conflicts of interest.

## References

[1] Phillips, M.L. & D.J. Kupfer . 2013 Bipolar disorder diagnosis: challenges and future directions. Lancet 381: 1663–1671. 23663952 10.1016/S0140-6736(13)60989-7 PMC5858935

[2] Bourne, C. , O. Aydemir , V. Balanza‐Martinez , *et al* 2013 Neuropsychological testing of cognitive impairment in euthymic bipolar disorder: an individual patient data meta‐analysis. Acta Psychiatr. Scand. 128: 149–162. 23617548 10.1111/acps.12133

[3] Ostergaard, S.D. , A. Bertelsen , J. Nielsen , *et al* 2013 The association between psychotic mania, psychotic depression and mixed affective episodes among 14,529 patients with bipolar disorder. J. Affect. Disord. 147: 44–50. 23122529 10.1016/j.jad.2012.10.005

[4] Vieta, E. & M. Valentí . 2013 Mixed states in DSM‐5: implications for clinical care, education, and research. J. Affect. Disord. 148: 28–36. 23561484 10.1016/j.jad.2013.03.007

[5] Broome, M. , K.E.A. Saunders , P.J. Harrison & S. Marwaha . 2015 Mood instability. Br. J. Psychiatry 207: 283–285. 26429679 10.1192/bjp.bp.114.158543 PMC4589661

[6] Faedda, G.L. , C. Marangoni , G. Serra , *et al* 2015 Precursors of bipolar disorders: a systematic literature review of prospective studies. J. Clin. Psychiatry 76: 614–624. 26035191 10.4088/JCP.13r08900

[7] Tsitsipa, E. & K. Fountoulakis . 2015 The neurocognitive functioning in bipolar disorder: a systematic review of data. Ann. Gen. Psychiatry 14: 42. 26628905 10.1186/s12991-015-0081-z PMC4666163

[8] Craddock, N. & M.J. Owen . 2010 The Kraepelinian dichotomy—going, going… but still not gone. Br. J. Psychiatry 196: 92–95. 20118450 10.1192/bjp.bp.109.073429 PMC2815936

[9] Miller, S. , B. Dell'Osso & T.A. Ketter . 2014 The prevalence and burden of bipolar depression. J. Affect. Disord. 169(Suppl. 1): S3–S11. 25533912 10.1016/S0165-0327(14)70003-5

[10] Di Florio, A. , N. Craddock & M. van den Bree . 2014 Alcohol misuse in bipolar disorder. A systematic review and meta‐analysis of comorbidity rates. Eur. Psychiatry 29: 117–124. 24075633 10.1016/j.eurpsy.2013.07.004

[11] Merikangas, K.R. , H.S. Akiskal , J. Angst , *et al* 2007 Lifetime and 12‐month prevalence of bipolar spectrum disorder in the National Comorbidity Survey replication. Arch. Gen. Psychiatry 64: 543–552. 17485606 10.1001/archpsyc.64.5.543 PMC1931566

[12] Parker, G. , S. McCraw , D. Hadzi‐Pavlovic & K. Fletcher . 2013 Costs of the principal mood disorders: a study of comparative direct and indirect costs incurred by those with bipolar I, bipolar II and unipolar disorders. J. Affect. Disord. 149: 46–55. 23141630 10.1016/j.jad.2012.10.002

[13] Pompili, M. , X. Gonda , G. Serafini , *et al* 2013 Epidemiology of suicide in bipolar disorders: a systematic review of the literature. Bipolar Disord. 15: 457–490. 23755739 10.1111/bdi.12087

[14] Hayes, J.F. , J. Miles , K. Walters , *et al* 2015 A systematic review and meta‐analysis of premature mortality in bipolar affective disorder. Acta Psychiatr. Scand. 131: 417–425. 25735195 10.1111/acps.12408 PMC4939858

[15] Chesney, E. , G.M. Goodwin & S. Fazel . 2014 Risks of all‐cause and suicide mortality in mental disorders: a meta‐review. World Psychiatry 13: 153–160. 24890068 10.1002/wps.20128 PMC4102288

[16] Kessing, L.V. , E. Vradi & P.K. Andersen . 2015 Life expectancy in bipolar disorder. Bipolar Disord. 17: 543–548. 25846854 10.1111/bdi.12296

[17] Cipriani, A. , J.R. Geddes , J. Higgins & G. Salanti . 2013 Conceptual and technical challenges in network meta‐analysis. Ann. Intern. Med. 159: 130–137. 23856683 10.7326/0003-4819-159-2-201307160-00008

[18] Mavridis, D. , M. Giannatsi , A. Cipriani & G. Salanti . 2015 A primer on network meta‐analysis with emphasis on mental health. Evid. Based Ment. Health 18: 40–46. 25908686 10.1136/eb-2015-102088 PMC11234956

[19] Geddes, J.R. & D.J. Miklowitz . 2013 Treatment of bipolar disorder. Lancet 381: 1672–1682. 23663953 10.1016/S0140-6736(13)60857-0 PMC3876031

[20] National Institute for Health and Clinical Excellence . 2014 Bipolar disorder: the assessment and management of bipolar disorder in adults, children and young people in primary and secondary care. NICE clinical guideline 185. http://guidance.nice.org.uk/CG185. Accessed March 23, 2016.

[21] Malhi, G.S. , D. Bassett , P. Boyce , *et al* 2015 Royal Australian and New Zealand College of Psychiatrists clinical practice guidelines for mood disorders. Aust. N.Z. J. Psychiatry 49: 1087–1206. 26643054 10.1177/0004867415617657

[22] Goodwin, G.M. & Consensus Group of the British Association for Psychopharmacology . 2009 Evidence‐based guidelines for treating bipolar disorder: revised third edition—recommendations from the British Association for Psychopharmacology. J. Psychopharmacol. 23: 346–388. 19329543 10.1177/0269881109102919

[23] Cipriani, A. , C. Barbui , G. Salanti , *et al* 2011 Comparative efficacy and acceptability of antimanic drugs in acute mania: a multiple‐treatments meta‐analysis. Lancet 378: 1306–1315. 21851976 10.1016/S0140-6736(11)60873-8

[24] Yildiz, A. , M. Nikodem , E. Vieta , *et al* 2015 A network meta‐analysis on comparative efficacy and all‐cause discontinuation of antimanic treatments in acute bipolar mania. Psychol. Med. 45: 299–317. 25036226 10.1017/S0033291714001305

[25] Frye, M.A. , M.L. Prieto , W.V. Bobo , *et al* 2014 Current landscape, unmet needs, and future directions for treatment of bipolar depression. J. Affect. Disord. 169(Suppl 1): S17–S23. 25533910 10.1016/S0165-0327(14)70005-9

[26] Kendall, T. , R. Morriss , E. Mayo‐Wilson , E. Marcus ; Guideline Development Group of the National Institute for Health and Care Excellence . 2014 Assessment and management of bipolar disorder: summary of updated NICE guidance. Br. Med. J. 349: g5673. 25258392 10.1136/bmj.g5673

[27] Taylor, D.M. , V. Cornelius , L. Smith & A.H. Young . 2014 Comparative efficacy and acceptability of drug treatments for bipolar depression: a multiple‐treatments meta‐analysis. Acta Psychiatr. Scand. 130: 452–469. 25283309 10.1111/acps.12343

[28] Durgam, S. , W. Earley , A. Lipschitz , *et al* 2016 An 8‐week randomized, double‐blind, placebo‐controlled evaluation of the safety and efficacy of cariprazine in patients with bipolar I depression. Am. J. Psychiatry 173: 271–281. 26541814 10.1176/appi.ajp.2015.15020164

[29] Geddes, J.R. , A. Gardiner , J. Rendell , *et al* 2016 Comparative evaluation of quetiapine plus lamotrigine versus quetiapine monotherapy (and folic acid versus placebo) in people with bipolar depression (CEQUEL): a 2 × 2 factorial randomised trial. Lancet Psychiatry 3: 31–39. 26687300 10.1016/S2215-0366(15)00450-2

[30] McIntyre, R.S. , J. Cucchiaro , A. Pikalov , *et al* 2015 Lurasidone in the treatment of bipolar depression with mixed (subsyndromal hypomanic) features: post hoc analysis of a randomized placebo‐controlled trial. J. Clin. Psychiatry 76: 398–405. 25844756 10.4088/JCP.14m09410

[31] Calabrese, J.R. , M.A. Frye , R. Yang , T.A. Ketter ; Armodafinil Treatment Trial Study Network . 2014 Efficacy and safety of adjunctive armodafinil in adults with major depressive episodes associated with bipolar I disorder: a randomized, double‐blind, placebo‐controlled, multicenter trial. J. Clin. Psychiatry 75: 1054–1061. 25099397 10.4088/JCP.13m08951

[32] Ketter, T.A. , R. Yang & M.A. Frye . 2015 Adjunctive armodafinil for major depressive episodes associated with bipolar I disorder. J. Affect. Disord. 181: 87–91. 25933099 10.1016/j.jad.2015.04.012

[33] McCloud, T.L. , C. Caddy , J. Jochim , *et al* 2015 Ketamine and other glutamate receptor modulators for depression in bipolar disorder in adults. Cochrane Database Syst. Rev. 9: CD011611. 26415966 10.1002/14651858.CD011611.pub2

[34] Schoeyen, H.K. , U. Kessler , O.A. Andreassen , *et al* 2015 Treatment‐resistant bipolar depression: a randomized controlled trial of electroconvulsive therapy versus algorithm‐based pharmacological treatment. Am. J. Psychiatry 172: 41–51. 25219389 10.1176/appi.ajp.2014.13111517

[35] Geddes, J.R. , S. Burgess , K. Hawton , *et al* 2004 Long‐term lithium therapy for bipolar disorder: systematic review and meta‐analysis of randomized controlled trials. Am. J. Psychiatry 161: 217–222. 14754766 10.1176/appi.ajp.161.2.217

[36] Geddes, J.R. , G.M. Goodwin , J. Rendell ; BALANCE Investigators . 2010 Lithium plus valproate combination therapy versus monotherapy for relapse prevention in bipolar I disorder (BALANCE): a randomised open‐label trial. Lancet 375: 385–395. 20092882 10.1016/S0140-6736(09)61828-6

[37] Cipriani, A. , C. Barbui , J. Rendell & J.R. Geddes . 2014 Clinical and regulatory implications of active run‐in phases in long‐term studies for bipolar disorder. Acta Psychiatr. Scand. 129: 328–342. 24289821 10.1111/acps.12223

[38] Miura, T. , H. Noma , T.A. Furukawa , *et al* 2014 Comparative efficacy and tolerability of pharmacological treatments in the maintenance treatment of bipolar disorder: a systematic review and network meta‐analysis. Lancet Psychiatry 1: 351–359. 26360999 10.1016/S2215-0366(14)70314-1

[39] Miziou, S. , E. Tsitsipa , S. Moysidou , *et al* 2015 Psychosocial treatment and interventions for bipolar disorder: a systematic review. Ann. Gen. Psychiatry 14: 19. 26155299 10.1186/s12991-015-0057-z PMC4493813

[40] McKnight, R.F. , M. Adida , K. Budge , *et al* 2012 Lithium toxicity profile: a systematic review and meta‐analysis. Lancet 379: 721–728. 22265699 10.1016/S0140-6736(11)61516-X

[41] Close, H. , J. Reilly , J.M. Mason , *et al* 2014 Renal failure in lithium‐treated bipolar disorder: a retrospective cohort study. PLoS One 26: e90169. 24670976 10.1371/journal.pone.0090169 PMC3966731

[42] Clos, S. , P. Rauchhaus , A. Severn , *et al* 2015 Long‐term effect of lithium maintenance therapy on estimated glomerular filtration rate in patients with affective disorders: a population‐based cohort study. Lancet Psychiatry 2: 1075–1083. 26453408 10.1016/S2215-0366(15)00316-8

[43] Kessing, L.V. , T.A. Gerds , B. Feldt‐Rasmussen , *et al* 2015 Use of lithium and anticonvulsants and the rate of chronic kidney disease: a nationwide population‐based study. JAMA Psychiatry 72: 1182–1191. 26535805 10.1001/jamapsychiatry.2015.1834

[44] Shine, B. , R.F. McKnight , L. Leaver & J.R. Geddes . 2015 Long‐term effects of lithium on renal, thyroid, and parathyroid function: a retrospective analysis of laboratory data. Lancet 386: 461–468. 26003379 10.1016/S0140-6736(14)61842-0

[45] Wesseloo, R. , A.M. Kamperman , T. Munk‐Olsen , *et al* 2016 Risk of postpartum relapse in bipolar disorder and postpartum psychosis: a systematic review and meta‐analysis. Am. J. Psychiatry 173: 117–127. 26514657 10.1176/appi.ajp.2015.15010124

[46] Diav‐Citrin, O. , S. Schechtman , E. Tahover , *et al* 2014 Pregnancy outcome following *in utero* exposure to lithium: a prospective, comparative, observational study. Am. J. Psychiatry 171: 785–794. 24781368 10.1176/appi.ajp.2014.12111402

[47] Bergink, V. & S.A. Kushner . 2014 Lithium during pregnancy. Am. J. Psychiatry 171: 712–715. 24980165 10.1176/appi.ajp.2014.14030409

[48] Bodén, R. , M. Lundgren , L. Brandt , *et al* 2012 Risks of adverse pregnancy and birth outcomes in women treated or not treated with mood stabilisers for bipolar disorder: population based cohort study. Br. Med. J. 345: e7085. 23137820 10.1136/bmj.e7085 PMC3493986

[49] Cipriani, A. , K. Hawton , S. Stockton & J.R. Geddes . 2013 Lithium in the prevention of suicide in mood disorders: updated systematic review and meta‐analysis. BMJ 346: f3646. 23814104 10.1136/bmj.f3646

[50] Gerhard, T. , D.P. Devenand , C. Huang , *et al* 2015 Lithium treatment and risk for dementia in adults with bipolar disorder: population‐based cohort study. Br. J. Psychiatry 207: 46–51. 25614530 10.1192/bjp.bp.114.154047

[51] Lan, C.C. , C.C. Liu , C.J. Lin , *et al* 2015 A reduced risk of stroke with lithium exposure in bipolar disorder: a population‐based retrospective cohort study. Bipolar Disord. 17: 705–714. 26394555 10.1111/bdi.12336

[52] Smith, E.G. , K.L. Austin , H.M. Kim , *et al* 2015 Mortality associated with lithium and valproate treatment of US Veterans Administration patients with mental disorders. Br. J. Psychiatry 207: 55–63. 25953891 10.1192/bjp.bp.113.138685

[53] Davis, J.M. , A. Rosenbaum , V. Shahinian & F.C. Brosius . 2015 Prevention of lithium‐associated renal failure: recent evidence. Lancet Psychiatry 2: 1045–1047. 26613842 10.1016/S2215-0366(15)00498-8

[54] Goodwin, G.M. 2015 The safety of lithium. JAMA Psychiatry 72: 1167–1169. 26536621 10.1001/jamapsychiatry.2015.2014

[55] Moore, J.L. & P. Aggarwal . 2012 Lamotrigine use in pregnancy. Exp. Opin. Pharmacother. 13: 1213–1216. 10.1517/14656566.2012.665875 22594849

[56] Correll, C. , J. Detraux , J. De Lepeliere & M. De Hert . 2015 Effects of antipsychotics, antidepressants and mood stabilizers on risk for physical diseases in people with schizophrenia, depression and bipolar disorder. World Psychiatry 14: 119–136. 26043321 10.1002/wps.20204 PMC4471960

[57] Tanoshima, M. , T. Kobayashi , R. Tanoshima , *et al* 2015 Risks of congenital malformations in offspring exposed to valproic acid *in utero*: a systematic review and cumulative meta‐analysis. Clin. Pharmacol. Ther. 98: 417–441. 26044279 10.1002/cpt.158

[58] Alda, M. 2015 Lithium in the treatment of bipolar disorder: pharmacology and pharmacogenetics. Mol. Psychiatry 20: 661–670. 25687772 10.1038/mp.2015.4 PMC5125816

[59] Berridge, M.J. 2014 Calcium signalling and psychiatric disease: bipolar disorder and schizophrenia. Cell Tissue Res. 357: 477–492. 24577622 10.1007/s00441-014-1806-z

[60] Tselnicker, I.F. , V. Tsemakhovich , I. Rishal , *et al* 2014 Dual regulation of G proteins and the G‐protein–activated K^+^ channels by lithium. Proc. Natl. Acad. Sci. U.S.A. 111: 5018–5023. 24639496 10.1073/pnas.1316425111 PMC3977261

[61] Hou, L. , U. Heilbronner , F. Degenhardt , *et al* 2016 Genetic variants associated with response to lithium treatment in bipolar disorder: a genome‐wide association study. Lancet. doi: 10.1016/S0140-6736(16)00143-4. 10.1016/S0140-6736(16)00143-4 PMC4814312 26806518

[62] Singh, N. , A.C. Halliday , J.M. Thomas , *et al* 2013 A safe lithium mimetic for bipolar disorder. Nat. Commun. 4: 1332. 23299882 10.1038/ncomms2320 PMC3605789

[63] Singh, N. , A.L. Sharpley , U.E. Emir , *et al* 2016 Effect of the putative lithium mimetic ebselen on brain *myo*‐inositol, sleep, and emotional processing in humans. Neuropsychopharmacology. doi: 10.1038/npp.2015.343. 10.1038/npp.2015.343 PMC4770517 26593266

[64] Schloesser, R.J. , K. Martinowich & H.K. Manji . 2012 Mood‐stabilizing drugs: mechanisms of action. Trends Neurosci. 35: 36–46. 22217451 10.1016/j.tins.2011.11.009

[65] Chiu, C.T. , Z. Wang , J.G. Hunsberger & D.M. Chuang . 2013 Therapeutic potential of mood stabilizers lithium and valproic acid: beyond bipolar disorder. Pharmacol. Rev. 65: 105–142. 23300133 10.1124/pr.111.005512 PMC3565922

[66] Brodie, M.J. 1992 Lamotrigine. Lancet 339: 1397–1400. 1350815 10.1016/0140-6736(92)91207-o

[67] Ketter, T.A. , H.K. Manji & R.M. Post . 2003 Potential mechanisms of action of lamotrigine in the treatment of bipolar disorders. J. Clin. Psychopharmacol. 23: 484–495. 14520126 10.1097/01.jcp.0000088915.02635.e8

[68] Goodwin, G.M. & J.R. Geddes . 2007 What is the heartland of psychiatry? Br. J. Psychiatry 191: 189–191. 17766755 10.1192/bjp.bp.107.036343

[69] Nutt, D. & G. Goodwin . 2011 ECNP summit on the future of CNS drug research in Europe 2011. Eur. Neuropsychopharmacol. 21: 495–499. 21684455 10.1016/j.euroneuro.2011.05.004

[70] Paykel, E.S. , R. Abbott , R. Morriss , *et al* 2006 Sub‐syndromal and syndromal symptoms in the longitudinal course of bipolar disorder. Br. J. Psychiatry 189: 118–123. 16880480 10.1192/bjp.bp.105.013870

[71] Bauer, M. , T. Glenn , P. Grof , *et al* 2010 Subsyndromal mood symptoms: a useful concept for maintenance studies of bipolar disorder. Psychopathology 43: 1–7. 19893338 10.1159/000255957

[72] Rush, A.J. , M.H. Trivedi , H.M. Ibrahim , *et al* 2003 The 16‐item Quick Inventory of Depressive Symptomatology (QIDS), clinician rating (QIDS‐C), and self‐report (QIDS‐SR): a psychometric evaluation in patients with chronic major depression. Biol. Psychiatry 54: 573–583. 12946886 10.1016/s0006-3223(02)01866-8

[73] Bilderbeck, A.C. , K.E.A. Saunders , G.D. Clifford , *et al* 2015 Daily and weekly mood ratings: relative contributions to the differentiation of bipolar disorder and borderline personality disorder. Bipolar Disord. 17(Suppl. 1): 129–130.

[74] Saunders, K.E.A. , A. Cipriani , J. Rendell , *et al* 2016 Oxford lithium trial (OxLith) of the early affective, cognitive, neural, biochemical effects of lithium carbonate in bipolar disorder: study protocol for a randomised controlled trial. Trials 17: 116. 26936776 10.1186/s13063-016-1230-7 PMC4776410

[75] Glenn, T. & S. Monteith . 2014 New measures of mental state and behavior based on data collected from sensors, smartphones, and the Internet. Curr. Psychiatry Rep. 16: 523. 25308392 10.1007/s11920-014-0523-3

[76] Faurholt‐Jepsen, M. , M. Frost , C. Ritz , *et al* 2015 Daily electronic self‐monitoring in bipolar disorder using smartphones—the MONARCA I trial: a randomized, placebo‐controlled, single‐blind, parallel group trial. Psychol. Med. 45: 2691–2704. 26220802 10.1017/S0033291715000410

[77] Hidalgo‐Mazzei, D. , A. Mateu , M. Reinares , *et al* 2015 Self‐monitoring and psychoeducation in bipolar patients with a smart‐phone application (SIMPLe) project: design, development and studies protocols. BMC Psychiatry 15: 52. 25884824 10.1186/s12888-015-0437-6 PMC4379950

[78] Nicholas, J. , M.E. Larsen , J. Proudfoot & H. Christensen . 2015 Mobile apps for bipolar disorder: a systematic review of features and content quality. J. Med. Internet Res. 17: e198. 26283290 10.2196/jmir.4581 PMC4642376

[79] Tourus, J. & A.C. Powell . 2015 Current research and trends in the use of smartphone applications for mood disorders. Internet Interv. 2: 169–173.

[80] Faurholt‐Jepsen, M. , K. Munkholm , M. Frost , *et al* 2016 Electronic self‐monitoring of mood using IT platforms in adult patients with bipolar disorder: a systematic review of the validity and evidence. BMC Psychiatry 16: 7. 26769120 10.1186/s12888-016-0713-0 PMC4714425

[81] Onnela, J.‐P. & S.L. Rauch . 2016 Harnessing smartphone‐based digital phenotyping to enhance behavioral and mental health. Neuropsychopharmacology. doi: 10.1038/npp.2016.7. 10.1038/npp.2016.7 PMC4869063 26818126

[82] Schwartz, S. , S. Schultz , A. Reider & E.F.H. Saunders . 2016 Daily mood monitoring of symptoms using smartphones in bipolar disorder: a pilot study assessing the feasibility of ecological momentary assessment. J. Affect. Disord. 191: 88–93. 26655117 10.1016/j.jad.2015.11.013 PMC4799837

[83] Saunders, K.E.A. , A.C. Bilderbeck , P. Panchal , *et al* 2015 Acceptability and tolerability of ambulatory monitoring in bipolar disorder: a patient perspective. Bipolar Disord. 17(Suppl. 1): 86.25046246

[84] Henry, C. , D. Van den Bulke , F. Bellivier , *et al* 2008 Affective lability and affect intensity as core dimensions of bipolar disorders during euthymic period. Psychiatry Res. 159: 1–6. 18295902 10.1016/j.psychres.2005.11.016

[85] Ortiz, A. , K. Bradler , J. Garnham , *et al* 2015 Nonlinear dynamics of mood regulation in bipolar disorder. Bipolar Disord. 17: 139–149. 25118155 10.1111/bdi.12246

[86] Bonsall, M.B. , S.M. Wallace‐Hadrill , J.R. Geddes , *et al* 2012 Nonlinear time‐series approaches in characterizing mood stability and mood instability in bipolar disorder. Proc. Biol. Sci. 279: 916–924. 21849316 10.1098/rspb.2011.1246 PMC3259919

[87] Moore, P.J. , M.A. Little , P.E. McSharry , *et al* 2014 Mood dynamics in bipolar disorder. Int. J. Bipolar Disord. 2: 11. 26092397 10.1186/s40345-014-0011-z PMC4452636

[88] Bonsall, M.B. , J.R. Geddes , E.A. Holmes & G.M. Goodwin . 2015 Bipolar disorder dynamics: affective instabilities, relaxation oscillations and noise. J. R. Soc. Interface 12: 20150670. 26577592 10.1098/rsif.2015.0670 PMC4685840

[89] Birmaher, B. , B.I. Goldstein , D.A. Axelson , *et al* 2013 Mood lability among offspring of parents with bipolar disorder and community controls. Bipolar Disord. 15: 253–263. 23551755 10.1111/bdi.12060 PMC3644367

[90] Hafeman, D.M. , J. Merranko , D. Axelson , *et al* 2016 Toward the definition of a bipolar prodrome: dimensional predictors of bipolar spectrum disorders in at‐risk youths. Am. J. Psychiatry. doi: 10.1176/appi.ajp.2015.15040414. 10.1176/appi.ajp.2015.15040414 PMC4930393 26892940

[91] Howes, O.D. , S. Lim , G. Theologos , *et al* 2011 A comprehensive review and model of putative prodromal features of bipolar affective disorder. Psychol. Med. 41: 1567–1577. 20836910 10.1017/S0033291710001790 PMC3687203

[92] Patel, R. , T. Lloyd , R. Jackson , *et al* 2015 Mood instability is a common feature of mental health disorders and is associated with poor clinical outcomes. BMJ Open 5: e007504. 10.1136/bmjopen-2014-007504 PMC4452754 25998036

[93] Strejilevich, S.A. , D.J. Martino , A. Murru , *et al* 2013 Mood instability and functional recovery in bipolar disorders. Acta Psychiatr. Scand. 128: 194–202. 23331090 10.1111/acps.12065

[94] Gershon, A. & P. Eidelman . 2015 Inter‐episode affective intensity and instability: predictors of depression and functional impairment in bipolar disorder. J. Behav. Ther. Exp. Psychiatry 46: 14–18. 25164093 10.1016/j.jbtep.2014.07.005 PMC4254202

[95] Solhan, M.B. , T.J. Trull , S. Jahng & P.K. Wood . 2009 Clinical assessment of affective instability: comparing EMA indices, questionnaire reports, and retrospective recall. Psychol. Assess. 21: 425–436. 19719353 10.1037/a0016869 PMC2864015

[96] Marwaha, S. , Z. He , M. Broome , *et al* 2014 How is affective instability defined and measured? A systematic review. Psychol. Med. 44: 1793–1808. 24074230 10.1017/S0033291713002407

[97] Broome, M.R. , Z. He , M. Iftikhar , *et al* 2015 Neurobiological and behavioural studies of affective instability in clinical populations: a systematic review. Neurosci. Biobehav. Rev. 51: 243–254. 25662294 10.1016/j.neubiorev.2015.01.021

[98] Beckmann, C.F. , M. DeLuca , J.T. Devlin & S.M. Smith . 2005 Investigations into resting‐state connectivity using independent component analysis. Phil. Trans. R. Soc. Lond. B Biol. Sci. 360: 1001–1013. 16087444 10.1098/rstb.2005.1634 PMC1854918

[99] Smith, S.M. , K.L. Miller , S. Moeller , *et al* 2012 Temporally‐independent functional modes of spontaneous brain activity. Proc. Natl. Acad. Sci. U.S.A. 109: 3131–3136. 22323591 10.1073/pnas.1121329109 PMC3286957

[100] Proudfoot, M. , M.W. Woolrich , A.C. Nobre & M.R. Turner . 2014 Magnetoencephalography. Pract. Neurol. 14: 336–343. 24647614 10.1136/practneurol-2013-000768 PMC4174130

[101] Brookes, M.J. , M. Woolrich , H. Luckhoo , *et al* 2011 Investigating the electrophysiological basis of resting state networks using magnetoencephalography. Proc. Natl. Acad. Sci. U.S.A. 108: 16783–16788. 21930901 10.1073/pnas.1112685108 PMC3189080

[102] Luckhoo, H. , J.R. Hale , M.G. Stokes , *et al* 2012 Inferring task‐related networks using independent component analysis in magnetoencephalography. Neuroimage 62: 530–541. 22569064 10.1016/j.neuroimage.2012.04.046 PMC3387383

[103] Baker, A.P. , M.J. Brookes , I.A. Rezek , *et al* 2014 Fast transient networks in spontaneous human brain activity. eLife 3: e01867. 24668169 10.7554/eLife.01867 PMC3965210

[104] Harmer, C.J. , P.J. Cowen & G.M. Goodwin . 2011 Efficacy markers in depression. J. Psychopharmacol. 25: 1148–1158. 20530590 10.1177/0269881110367722

[105] Warren, M.B. , A. Pringle & C.J. Harmer . 2015 A neurocognitive model for understanding treatment action in depression. Philos. Trans. R. Soc. Lond. B Biol. Sci. 370: 20140213. 26240428 10.1098/rstb.2014.0213 PMC4528825

[106] Phillips, M.L. & H.A. Swartz . 2014 A critical appraisal of neuroimaging studies of bipolar disorder: toward a new conceptualization of underlying neural circuitry and a road map for future research. Am. J. Psychiatry 171: 829–843. 24626773 10.1176/appi.ajp.2014.13081008 PMC4119497

[107] Craddock, N. & P. Sklar . 2013 Genetics of bipolar disorder. Lancet 381: 1654–1662. 23663951 10.1016/S0140-6736(13)60855-7

[108] Shinozaki, G. & J.B. Potash . 2014 New developments in the genetics of bipolar disorder. Curr. Psychiatry Rep. 16: 493. 25194313 10.1007/s11920-014-0493-5

[109] Mühleisen, T.W. , M. Leber , T.G. Schulze , *et al* 2014 Genome‐wide association study reveals two new risk loci for bipolar disorder. Nat. Commun. 11: 3339. 24618891 10.1038/ncomms4339

[110] Green, E.K. , E. Rees , J.T.R. Walters , *et al* 2016 Copy number variation in bipolar disorder. Mol. Psychiatry 21: 89–93. 25560756 10.1038/mp.2014.174 PMC5038134

[111] Hasler, G. & A. Wolf . 2015 Toward stratified treatments for bipolar disorders. Eur. Neuropsychopharmacol. 25: 283–294. 25595029 10.1016/j.euroneuro.2014.12.006

[112] Plenge, R.M. , E.M. Scolnick & D. Altshuler . 2013 Validating therapeutic targets through human genetics. Nat. Rev. Drug Discov. 12: 581–594. 23868113 10.1038/nrd4051

[113] Harrison, P.J. 2015 Recent genetic findings in schizophrenia and their therapeutic relevance. J. Psychopharmacol. 29: 85–96. 25315827 10.1177/0269881114553647 PMC4361495

[114] Nelson, M.R. , H. Tipney , J.L. Painter , *et al* 2015 The support of human genetic evidence for approved drug indications. Nat. Genet. 47: 856–860. 26121088 10.1038/ng.3314

[115] Papassotiropoulos, A. & D.J. de Quervain . 2015 Failed drug discovery in psychiatry: time for human genome‐guided solutions. Trends Cogn. Sci. 19: 183–187. 25727774 10.1016/j.tics.2015.02.002

[116] Harrison, P.J. 2016 Molecular neurobiological clues to the pathogenesis of bipolar disorder. Curr. Opin. Neurobiol. 36: 1–6. 26210959 10.1016/j.conb.2015.07.002 PMC4779149

[117] Warsh, J.J. , S. Andreopoulos & P.P. Li . 2004 Role of intracellular calcium signaling in the pathophysiology and pharmacotherapy of bipolar disorder: current status. Clin. Neurosci. Res. 4: 201–213.

[118] Bhat, S. , D.T. Dao , C.E. Terrillion , *et al* 2012 CACNA1C (Ca_v_1.2) in the pathophysiology of psychiatric disease. Prog. Neurobiol. 99: 1–14. 22705413 10.1016/j.pneurobio.2012.06.001 PMC3459072

[119] McCarthy, M.J. , M.J. Le Roux , H. Wei , *et al* 2016 Calcium channel genes associated with bipolar disorder modulate lithium's amplification of circadian rhythms. Neuropharmacology 101: 439–448. 26476274 10.1016/j.neuropharm.2015.10.017 PMC4681663

[120] Ament, S.A. , S. Szelinger , G. Glusman , *et al* 2015 Rare variants in neuronal excitability genes influence risk for bipolar disorder. Proc. Natl. Acad. Sci. U.S.A. 112: 3576–3581. 25730879 10.1073/pnas.1424958112 PMC4371952

[121] Levy, N.A. & P.G. Janicak . 2000 Calcium channel antagonists for the treatment of bipolar disorder. Bipolar Disord. 2: 108–119. 11252650 10.1034/j.1399-5618.2000.020204.x

[122] Cipriani, A. , K. Saunders , M.‐J. Attenburrow , *et al* 2016 Calcium channel antagonists in bipolar disorder. A systematic review of calcium channel antagonists in bipolar disorder and some considerations for their future development. Mol. Psychiatry. In press. 10.1038/mp.2016.86 PMC5030455 27240535

[123] Ostacher, M.J. , D.V. Iosifescu , A. Hay , *et al* 2014 Pilot investigation of isradipine in the treatment of bipolar depression motivated by genome‐wide association. Bipolar Disord. 2: 199–203. 24372835 10.1111/bdi.12143

[124] Striessnig, J. , A. Pinggera , G. Kaur , *et al* 2014 L‐type Ca^2+^ channels in heart and brain. Wiley Interdiscip. Rev. Membr. Transp. Signal. 3: 15–38. 24683526 10.1002/wmts.102 PMC3968275

[125] Hopkins, A.L. & C.R. Groom . 2002 The druggable genome. Nat. Rev. Drug Discov. 1: 727–730. 12209152 10.1038/nrd892

[126] Rasband, M.N. 2010 The axon initial segment and the maintenance of neuronal polarity. Nat. Rev. Neurosci. 11: 552–562. 20631711 10.1038/nrn2852

[127] Smith, K.R. , K.J. Kopeikina , J.M. Fawcett‐Patel , *et al* 2014 Psychiatric risk factor ANK3/ankyrin‐G nanodomains regulate the structure and function of glutamatergic synapses. Neuron 84: 399–415. 25374361 10.1016/j.neuron.2014.10.010 PMC4223651

[128] Durak, O. , F.C. de Anda , K.K. Singh , *et al* 2015 Ankyrin‐G regulates neurogenesis and Wnt signaling by altering the subcellular localization of β‐catenin. Mol. Psychiatry 20: 388–397. 24821222 10.1038/mp.2014.42 PMC4231016

[129] Rueckert, E.H. , D. Barker , D. Ruderfer , *et al* 2013 *Cis*‐acting regulation of brain‐specific ANK3 gene expression by a genetic variant associated with bipolar disorder. Mol. Psychiatry 18: 922–929. 22850628 10.1038/mp.2012.104 PMC3856665

[130] Cross‐Disorder Group of the Psychiatric Genomics Consortium . 2013 Identification of risk loci with shared effects on five major psychiatric disorders: a genome‐wide analysis. Lancet 381: 1371–1379. 23453885 10.1016/S0140-6736(12)62129-1 PMC3714010

[131] Craddock, N. , L. Jones , I.R. Jones , *et al*; Wellcome Trust Case Control Consortium (WTCCC). 2010 Strong genetic evidence for a selective influence of GABA_A_ receptors on a component of the bipolar disorder phenotype. Mol. Psychiatry 15: 146–153. 19078961 10.1038/mp.2008.66 PMC3967096

[132] Goes, F.S. , M.L. Hamshere , F. Seifuddin , *et al*; Bipolar Genome Study (BIGS). 2012 Genome‐wide association of mood‐incongruent psychotic bipolar disorder. Transl. Psychiatry 2: e180. 23092984 10.1038/tp.2012.106 PMC3565814

[133] Greenwood, T.A. , Bipolar Genome Study (BiGS) Consortium & J.R. Kelsoe . 2013 Genome‐wide association study of irritable vs. elated mania suggests genetic differences between clinical subtypes of bipolar disorder. PLoS One 8: e53804. 23326512 10.1371/journal.pone.0053804 PMC3542199

[134] Insel, T. , B. Cuthbert , M. Garvey , *et al* 2010 Research domain criteria (RDoC): toward a new classification framework for research on mental disorders. Am. J. Psychiatry 167: 748–751. 20595427 10.1176/appi.ajp.2010.09091379

[135] Lee, R.S. , D.F. Hermens , J. Scott , *et al* 2014 A meta‐analysis of neuropsychological functioning in first‐episode bipolar disorders. J. Psychiatr. Res. 57: 1–11. 25016347 10.1016/j.jpsychires.2014.06.019

[136] Martinez‐Aran, A. & E. Vieta . 2015 Cognition as a target in schizophrenia, bipolar disorder and depression. Eur. Neuropsychopharmacol. 25: 151–157. 25661911 10.1016/j.euroneuro.2015.01.007

[137] Harvey, A.G. 2008 Sleep and circadian rhythms in bipolar disorder: seeking synchrony, harmony, and regulation. Am. J. Psychiatry 165: 820–829. 18519522 10.1176/appi.ajp.2008.08010098

[138] Levenson, J.C. , D.A. Axelson , J. Merranko , *et al* 2015 Differences in sleep disturbances among offspring of parents with and without bipolar disorder: association with conversion to bipolar disorder. Bipolar Disord. 17: 836–848. 26547512 10.1111/bdi.12345 PMC4734929

[139] Ritter, P.S. , M. Höfler , H.U. Wittchen , *et al* 2015 Disturbed sleep as risk factor for the subsequent onset of bipolar disorder—data from a 10‐year prospective‐longitudinal study among adolescents and young adults. J. Psychiatr. Res. 68: 76–82. 26228404 10.1016/j.jpsychires.2015.06.005

[140] Cretu, J.B. , J.L. Culver , K.C. Goffin , *et al* 2016 Sleep, residual symptoms, and time to relapse in recovered patients with bipolar disorder. J. Affect. Disord. 190: 162–166. 26519636 10.1016/j.jad.2015.09.076

[141] Pinho, M. , M. Sehmbi , L. Cudney , *et al* 2016 The association between biological rhythms, depression, and functioning in bipolar disorder: a large multi‐center study. Acta Psychiatr. Scand. 133: 102–108. 10.1111/acps.12442 26010130

[142] Byrne, E.M. , P.R. Gehrman , S.E. Medland , *et al*; Chronogen Consortium . 2013 A genome‐wide association study of sleep habits and insomnia. Am. J. Med. Genet. B Neuropsychiatr. Genet. 162B: 439–451. 23728906 10.1002/ajmg.b.32168 PMC4083458

[143] Parsons, M.J. , K.J. Lester , N.L. Barclay , *et al* 2013 Replication of genome‐wide association studies (GWAS) loci for sleep in the British G1219 cohort. Am. J. Med. Genet. B Neuropsychiatr. Genet. 162B: 431–438. 23780892 10.1002/ajmg.b.32106

[144] Heck, A. , M. Fastenrath , S. Ackermann , *et al* 2014 Converging genetic and functional brain imaging evidence links neuronal excitability to working memory, psychiatric disease, and brain activity. Neuron 81: 1203–1213. 24529980 10.1016/j.neuron.2014.01.010 PMC4205276

[145] Heck, A. , M. Fastenrath , D. Coynel , *et al* 2015 Genetic analysis of association between calcium signaling and hippocampal activation, memory performance in the young and old, and risk for sporadic Alzheimer disease. JAMA Psychiatry 72: 1029–1036. 26332608 10.1001/jamapsychiatry.2015.1309 PMC5291164

[146] Viswanath, B. , S.P. Jose , A. Squassina , *et al* 2015 Cellular models to study bipolar disorder: a systematic review. J. Affect. Disord. 15: 36–50. 26070045 10.1016/j.jad.2015.05.037

[147] Andreazza, A.C. & L.T. Young . 2015 The neurobiology of bipolar disorder: identifying targets for specific agents and synergies for combination treatment. Int. J. Neuropsychopharmacol. 17: 1039–1052. 23449044 10.1017/S1461145713000096

[148] Chen, H.M. , C.J. DeLong , M. Bame , *et al* 2014 Transcripts involved in calcium signaling and telencephalic neuronal fate are altered in induced pluripotent stem cells from bipolar disorder patients. Transl. Psychiatry 4: e375. 25116795 10.1038/tp.2014.12 PMC3966040

[149] Wang, J.L. , S.M. Shamah , A.X. Sun , *et al* 2014 Label‐free, live optical imaging of reprogrammed bipolar disorder patient‐derived cells reveals a functional correlate of lithium responsiveness. Transl. Psychiatry 4: e428. 25158003 10.1038/tp.2014.72 PMC4150245

[150] Madison, J.M. , F. Zhou , A. Nigam , *et al* 2015 Characterization of bipolar disorder patient‐specific induced pluripotent stem cells from a family reveals neurodevelopmental and mRNA expression abnormalities. Mol. Psychiatry 20: 703–717. 25733313 10.1038/mp.2015.7 PMC4440839

[151] Mertens, J. , Q.‐W. Wang , Y. Kim , *et al*; Pharmacogenomics of Bipolar Disorder Study . 2015 Differential responses to lithium in hyperexcitable neurons from patients with bipolar disorder. Nature 527: 95–99. 26524527 10.1038/nature15526 PMC4742055

[152] Yoshimizu, T. , J.Q. Pan , A.E. Mungenast , *et al* 2015 Functional implications of a psychiatric risk variant within CACNA1C in induced human neurons. Mol. Psychiatry 20: 162–169. 25403839 10.1038/mp.2014.143 PMC4394050

[153] Bigos, K.L. , V.S. Mattay , J.H. Callicott , *et al* 2010 Genetic variation in CACNA1C affects brain circuitries related to mental illness. Arch. Gen. Psychiatry 67: 939–945. 20819988 10.1001/archgenpsychiatry.2010.96 PMC3282053

[154] Malhi, G.S. & J.R. Geddes . 2014 Carving bipolarity using a lithium sword. Br. J. Psychiatry 205: 337–339. 25368357 10.1192/bjp.bp.114.148072

[155] Tighe, S.K. , P.B. Mahon & J.B. Potash . 2011 Predictors of lithium response in bipolar disorder. Ther. Adv. Chronic Dis. 2: 209–226. 23251751 10.1177/2040622311399173 PMC3513882

[156] Harrison, P.J. , M.Z. Cader & J.R. Geddes . 2016 Reprogramming psychiatry: stem cells and bipolar disorder. Lancet 387: 823–825. 26972063 10.1016/S0140-6736(16)00235-X

[157] Kaiser, T. & G. Feng . 2015 Modeling psychiatric disorders for developing effective treatments. Nat. Med. 21: 979–988. 26340119 10.1038/nm.3935 PMC4886231

[158] Einat, H. 2007 Different behaviors and different strains: potential new ways to model bipolar disorder. Neurosci. Biobehav. Rev. 31: 850–857. 17307253 10.1016/j.neubiorev.2006.12.001

[159] Cosgrove, V.E. , J.R. Kelsoe & T. Suppes . 2016 Toward a valid animal model of bipolar disorder: how the research domain criteria help bridge the clinical–basic science divide. Biol. Psychiatry 79: 62–70. 26531027 10.1016/j.biopsych.2015.09.002

[160] Han, K. , J.L. Holder , C.P. Schaaf , *et al* 2013 SHANK3 overexpression causes manic‐like behaviour with unique pharmacogenetic properties. Nature 503: 72–77. 24153177 10.1038/nature12630 PMC3923348

[161] Leussis, M.P. , E.M. Berry‐Scott , M. Saito , *et al* 2013 The ANK3 bipolar disorder gene regulates psychiatric‐related behaviors that are modulated by lithium and stress. Biol. Psychiatry 73: 683–690. 23237312 10.1016/j.biopsych.2012.10.016

[162] Sidor, M.M. , S.M. Spencer , K. Dzirasa , *et al* 2015 Daytime spikes in dopaminergic activity drive rapid mood‐cycling in mice. Mol. Psychiatry 20: 1406–1419. 25560763 10.1038/mp.2014.167 PMC4492925

[163] Chung, S. , E.J. Lee , S. Yun , *et al* 2014 Impact of circadian nuclear receptor REV‐ERBα on midbrain dopamine production and mood regulation. Cell 157: 858–868. 24813609 10.1016/j.cell.2014.03.039

[164] McClung, C.A. 2011 Circadian rhythms and mood regulation: insights from pre‐clinical models. Eur. Neuropsychopharmacol. 21(Suppl. 4): S683–S693. 21835596 10.1016/j.euroneuro.2011.07.008 PMC3179573

